# Clinical and Epidemiological Characteristics of Multiple Sclerosis in a Peruvian Cohort: A Retrospective Study Across Three Lima Hospitals

**DOI:** 10.3390/neurolint18090160

**Published:** 2026-08-24

**Authors:** Ana Cruz Cruz, Julio Perez-Villegas, Eduardo Alvarado, Edward Smith, Ivan Dueñas-Pacheco

**Affiliations:** 1 Hospital Nacional Alberto Sabogal-Essalud, Lima 07011, Peru; anacruz.cruz@yahoo.es; 2 Neurological Unit, National Hospital Dos de Mayo, Lima 15001, Peru; julioperezvillegas@yahoo.es; 3 Roche Farma, Lima 15047, Peru; eduardo.alvarado.ea1@roche.com; 4 Servicio de Neurología, Hospital Nacional María Auxiliadora, Lima 15801, Peru; ivandpneuro@hotmail.com

**Keywords:** multiple sclerosis, Peru, epidemiology

## Abstract

**Background/Objectives**: To describe the demographic, clinical, and epidemiological characteristics of a cohort of patients diagnosed with Multiple Sclerosis (MS) in Lima, Peru. **Methods**: A descriptive, retrospective cohort study conducted across three major hospitals in Lima. We included adult patients diagnosed with MS in any of its clinical phenotypes—Clinically Isolated Syndrome (CIS), Relapsing-Remitting MS (RRMS), Secondary Progressive MS (SPMS), and Primary Progressive MS (PPMS)—who were followed between 2007 and 2017. **Results**: A total of 103 patients were identified. The majority were women (58.3%), with a median age at onset of 38.4 years (Interquartile Range [IQR]: 28.9–46.5). The most common clinical phenotype at onset was RRMS (90.3%), which remained the most prevalent phenotype at the current evaluation (79.6%). Twelve patients (12.9%) progressed from RRMS to SPMS by their last control visit. The most frequent symptoms at onset were limb weakness (72.8%), sensory symptoms (72.8%), and blurred vision (42.0%). At the last evaluation, the most frequent symptoms were limb weakness (78.6%), sensory symptoms (71.8%), and spasticity (40.2%). The most common initial treatment was interferon-beta (44.7%), while two patients (1.9%) initiated therapy with a highly effective disease-modifying drug (DMD), Ocrelizumab. The most recent treatment reported remained interferon-beta (33.9%), but 12 patients (11.7%) were currently receiving Ocrelizumab. **Conclusions**: This study describes clinical characteristics at onset and during follow-up that are largely similar to those reported in international series. However, we found no evidence of improved disease evolution, a finding potentially attributable to the heterogeneity of treatment and management strategies employed across the cohort.

## 1. Introduction

The Multiple Sclerosis (MS) is a chronic, autoimmune disease of the central nervous system (CNS), with more than 2 million prevalent cases of MS globally in 2016, which corresponded to a 10.4% increase in the age-standardized prevalence since 1990 [1], and highest prevalence rates are traditionally reported in Northern Europe and North America [2]. Conversely, the disease burden in Peru remains relatively low, with an estimated prevalence of 7.7 per 100,000 individuals [3]. Despite this lower regional prevalence, MS carries a significant public health impact globally, as it is the most frequent cause of non-traumatic disabling neurological disease affecting young adults [4].

The hallmark of MS pathology is the development of perivenular inflammatory lesions characterized by infiltrating CD8+ T cells, which ultimately leads to demyelination and axonal loss (plaque formation). While the initiating pathological mechanisms are heterogenous, the effector mechanisms become similar in the later stages of the disease. The clinical manifestations of MS are diverse and include acute or subacute motor weakness, unilateral visual loss (optic neuritis), sensory loss in the limbs or face, diplopia, gait disturbance, Lhermitte’s sign, vertigo, and bladder dysfunction [5]. MS is clinically classified into four main phenotypes: Clinically Isolated Syndrome (CIS), Relapsing-Remitting MS (RRMS), Secondary Progressive MS (SPMS), and Primary Progressive MS (PPMS). RRMS is reported as the most frequent initial phenotype, although 5–15% of patients present with PPMS, and most RRMS patients transition to SPMS after a period of 10 to 15 years [4]. The exact etiology of MS remains unclear, with evidence suggesting a complex interplay between various genetic and environmental risk factors [5,6]. Consistent with other autoimmune disorders, MS is more prevalent in women than in men, and is typically diagnosed between the ages of 20 and 40 years. Other identified risk factors include low vitamin D levels, smoking history, obesity, and prior Epstein-Barr virus infection [7].

Evidence characterizing the clinical presentation, demographics, and treatment response of MS patients in Latin America, and specifically in Peru [8,9,10,11,12,13,14], remain sparse. Recognizing the recent development of internationally adapted diagnostic criteria that incorporate the experience of diverse populations (including pediatric, Asian, and Latin American cohorts) [15], there is a critical need to contribute local epidemiological data. Therefore, this study aims to provide a comprehensive description of the demographic, clinical, and epidemiological characteristics of a patient series diagnosed with MS across three major hospitals in Lima, Peru.

## 2. Materials and Methods

This was a descriptive retrospective cohort study examining Peruvian patients diagnosed with Multiple Sclerosis (MS). The cohort was identified by reviewing medical records across three major hospitals located in Lima, Peru: Hospital Nacional Dos de Mayo, Hospital Nacional Alberto Sabogal Sologuren, and Hospital Nacional María Auxiliadora. Data were collected over a 10-year period, spanning from 1 January 2007, to 31 December 2017.

All clinical charts within the selected hospitals were reviewed against the following inclusion and exclusion criteria. Adult patients with a confirmed diagnosis of MS (any clinical phenotype) who were diagnosed using the contemporary consensus criteria at the time of evaluation (the 2005 McDonald criteria for patients diagnosed prior to 2010, and the 2010 McDonald criteria for those diagnosed from 2010 onward) and who had documented data available for at least 2 years within the respective institution were included. Demographic data (age, sex), clinical characteristics (year of diagnosis, smoking status, initial treatment, current treatment, clinical features at onset, and clinical features at the time of the current evaluation) were extracted from the electronic and/or paper medical records of each included patient. Data collection was performed using a standardized case record form (CRF).

For the statistical analysis, frequencies and percentages were used to describe categorical variables. Continuous variables were reported as medians and the interquartile range (IQR). All statistical computations were performed using the STATA statistical software package, version 15.1 (StataCorp, College Station, TX, USA).

## 3. Results

A total of 103 patients with Multiple Sclerosis (MS) were identified across the three hospitals. The majority of the cohort was female (*n* = 60, 58.3%). The median age at disease onset was 38.4 years (Interquartile Range [IQR]: 28.9–46.5), with the age range spanning from 17.7 years to 70.9 years. The most common clinical phenotype at onset was RRMS (*n* = 93, 90.3%), which remained the most frequent phenotype at the last recorded clinical evaluation (*n* = 82, 79.6%). Twelve patients (12.9%) demonstrated progression from RRMS to SPMS by their last follow-up visit. No history of Epstein-Barr virus infection in early life was reported in this cohort. Cerebrospinal fluid (CSF) analysis revealed abnormal IgG oligoclonal band (OCB) positivity in 34 cases (63.4%). The general characteristics of the population at disease onset and the current evaluation are detailed in Table 1.

The most frequent symptoms reported at disease onset were limb weakness (*n* = 75/103, 72.8%), sensory symptoms (*n* = 75/103, 72.8%), and blurred vision (*n* = 42/100, 42.0%). At the time of the last evaluation, the most frequent symptoms were limb weakness (*n* = 81/103, 78.6%), sensory symptoms (*n* = 74/103, 71.8%), and spasticity (*n* = 61/102, 40.2%). The full distribution of symptoms is presented in Table 2.

The median relapse rate was 0.6 per year (IQR: 0.3–1.0). Regarding hospitalizations, one hospitalization per year was the most frequent frequency (*n* = 43/88, 48.9%), with a stay of 5 days being the most common duration (*n* = 19/89, 21.4%), largely driven by the administration of intravenous corticosteroids. Thirty-seven patients (35.9%) reported engaging in physical therapy.

Analysis of the Expanded Disability Status Scale (EDSS) showed that the median change between the EDSS score at onset and at the last evaluation was 0 (IQR: 0–0). When stratified by follow-up duration, patients with less than 10 years of follow-up maintained a median EDSS change of 0 (IQR: 0–0). However, those with 10 or more years of follow-up demonstrated a median reduction in EDSS score (median −1, IQR: −1 to 0). EDSS scores at onset and last evaluation, stratified by follow-up duration, are detailed in Table 1.

Only 51 patients (49.5%) reported receiving any type of Disease-Modifying Therapy (DMT) at disease onset. The most frequent initial treatment was interferon beta (*n* = 46/103, 44.7%), and this remained the most frequently recorded current treatment (*n* = 35/103, 34.0%). Treatment duration was heterogeneous, with a median length of 5 years (IQR: 2.3–8.8), ranging from a minimum of 4 months to a maximum of 20 years. Patients primarily stayed on their initial first-line treatment unless they met criteria for treatment failure (defined as unmitigated clinical relapses or confirmed radiological progression).

Only three patients (2.8%) initiated therapy with a high-efficacy DMT at onset: Ocrelizumab (*n* = 2, 1.9%) and Fingolimod (*n* = 1, 0.9%). By the time of the last evaluation, 18 patients (17.5%) were registered as receiving a high-efficacy DMT, though all patients receiving these therapies, except for one on Fingolimod, were managed within the context of clinical trials. The remaining distribution of treatments is outlined in Table 3. The vast majority of patients (*n* = 91, 88.4%) reported adherence to their prescribed treatment. Regarding functional impact, 56 patients (54.4%) reported unemployment due to MS. Among those who required a caregiver (*n* = 43), the majority were non-professional caregivers (*n* = 41, 95.4%). The median current EDSS score was 4 (IQR: 3–5.5) for patients reporting unemployment and 5 (IQR: 3.5–5.5) for those requiring a caregiver.

## 4. Discussion

This 10-year retrospective study across three major referral hospitals provides a robust description of the MS population in Lima, Peru. Our results generally align with established international literature, specifically concerning the female predominance and the high frequency of the RRMS phenotype at both onset and follow-up [5,16]. Consistent with other reports, the PPMS phenotype was the second most common form encountered. However, we found a notably low frequency of reported smoking (7.3%) compared to global cohorts. This finding may reflect both the lower prevalence of smoking in Peruvian population and the inherent limitations of retrospective chart review, where non-standardized or absent documentation likely leads to an underestimation of this exposure. Furthermore, key established environmental and serological risk factors, such as obesity and prior exposure to Epstein-Barr virus (EBV), could not be assessed due to the absence of systematic recording in the medical charts.

The most frequent initial treatment in patients with MS in this cohort was found to be interferon beta (44.7%). These patients appear to show no change in their EDSS score when assessed at their last visit; however, the difference in follow-up length and disease stage at the final evaluation creates a high risk of bias in these results. Regarding interferon beta, a series of 20 patients reported in 2005 at a hospital in Lima demonstrates local experience with its use, finding 75% of patients relapse-free at the end of treatment. However, this evaluation was observational and carries a high risk of bias and other threats to the validity of the results [12]. Similarly, another study conducted in Peru (*n* = 12) found that the early use of interferon in these patients showed no recurrence of optic neuritis (something found in 42% of our series), suggesting that early treatment can reduce the impact of such complications [17].

For patients with RRMS who exhibit non-aggressive disease onset or who are not identified as high-risk for initial treatment failure, the latest recommendations advise initiating therapy with conventional disease-modifying agents such as interferon beta, glatiramer acetate, or teriflunomide [6]. It is important to emphasize that early initiation of disease-modifying therapies in patients with RRMS is important to reduce the impact of disability, reduce relapses, and reduce inflammation and brain damage [18,19,20]. There is evidence of relapse reductions of 46–47% with ocrelizumab, 28–34% with interferon beta, 29% with glatiramer acetate, 54% with fingolimod, and 31% with teriflunomide [6]. In our study, where over 50% of patients lost their jobs due to MS and 41.7% require caregivers, we found that only 49.5% started any treatment at the onset of the disease. Therefore, it is crucial to emphasize the importance of initiating early therapy with disease-modifying therapies to alleviate the course of the disease and reduce the frequency of relapses.

To fulfill the diagnostic requirements of the McDonald criteria, every patient in this three-center cohort underwent at least two magnetic resonance imaging (MRI) protocols (brain and/or spinal cord) to formally confirm dissemination in space (DIS) and dissemination in space (DIT) while systematically ruling out alternative structural or inflammatory mimicking pathologies. However, because this study relies on a multi-center, 10-year retrospective chart review across differing institutional radiology platforms, highly granular, standardized quantitative neuroimaging parameters—such as precise T2/FLAIR lesion counts, baseline gadolinium-enhancing lesion volumes, or brain atrophy metrics—were not uniformly structured within the available historical medical records. While the lack of an integrated, quantitative neuroimaging database represents an acknowledged limitation of our retrospective design, it reflects a common real-world challenge in resource-limited public health settings where qualitative diagnostic confirmation takes clinical precedence over standardized volumetric tracking.

To better understand how the epidemiological and clinical landscapes of MS in Peru compare to broader trends, our findings can be contextualized within the existing local literature and standard international benchmarks: first, our cohort demonstrated a female predominance of 58.3%, similar with local and global reports [1,9,10]. Second, the median age at onset in our series was 38.5 years, aligning closely with similar Peruvian reports (35.0–33.7) [9,10]. The literature describing the status of MS in Peru remains sparse, underscoring the necessity of regional descriptive studies. Previous local investigations have established a baseline for the disease, with a 2009 study reporting a national prevalence of 7.7 per 100,000 persons [3], a figure subsequently refined by a capture-recapture analysis that estimated a point prevalence of 9.1 per 100,000 persons [10]; furthermore, this last study found RRMS as the predominant phenotype (79.3%) and highlighted interferon beta as the most frequent treatment (40.7%) during that period [10]. Our findings align strongly with these established patterns regarding phenotype distribution and symptomology. Earlier case series from Lima, including studies between 1993 and 2004 (*n* = 55) and work from the Hospital Alberto Sabogal (*n* = 24), consistently reported RRMS as the most common form, with frequent initial involvement being motor and sensory deficits, decreased visual acuity, and optic neuritis [14,21]. Furthermore, a prior analysis of 13 patients at the National Institute of Neurological Sciences (INCN) found a frequency of depression (38.5%) and cognitive impairment (1.45%) [11], observations that closely parallel our current results (depression 34.7%; cognitive dysfunction 25.3%).

However, our cohort contributes key important discrepancies. While previous local reports described onset ages ranging from 33 to 39 years, a broader series compiled from literature reported a mean age at diagnosis of 27 years [22], which is notably lower than the median of 38.5 years found in our study. More recent work from the INCN (*n* = 121) detailed the high frequency of myelitis (35%) and brainstem syndrome (25%) at onset for RRMS patients, with brainstem syndrome identified as a predictor for earlier diagnosis [9]. Our large, multi-center cohort reinforces the consistency of the clinical picture across different Lima institutions while providing a more robust median age of onset and current data on the socio-economic burden and DMT utilization.

The inherent limitations of this study require cautious interpretation. As a descriptive study relying on a convenience sample from three referral hospitals, our findings may not be generalizable to the entire MS population across Peru. Nevertheless, the size of our cohort (*n* = 103) represents one of the largest published series to date from Peru and includes data from major specialized centers. Data incompleteness represents a further limitation, common in chart reviews, with missing variables, including EBV exposure, serum Vitamin D levels, and specific genetic polymorphisms (e.g., HLA-DRB1*15:01). The lack of routine, comprehensive neuropsychological evaluations likely lead to an underestimation of cognitive impairment, a pervasive issue reported in MS literature globally (e.g., 28% impairment in one large German cohort [23]). Furthermore, we did not assess quality of life, which is known to be significantly impaired in MS patients compared to other chronic diseases and is critical for evaluating therapeutic success [24,25].

This study provides one of the largest available descriptions of the clinical and demographic characteristics of Multiple Sclerosis (MS) in Peru, confirming general similarities to international series but starkly highlighting the significant social and economic disability burden observed within this cohort. Given the substantial functional loss and high dependency rates reported, our results require a critical reassessment of the current therapeutic approach to ensure the early and sustained use of highly effective disease-modifying therapies (DMTs). While our findings underscore the clinical reality in a resource-limited setting, the high risk of bias inherent to the retrospective design demands further investigation. Implementation of prospective cohort studies employing standardized measurement tools and consistent follow-up protocols to robustly characterize, monitor, and optimize the management of MS patients across the Peruvian healthcare system are urgently needed.

## Figures and Tables

**Table 1:** Patients’ characteristics.

Characteristic	*n* = 103
Women (%)	60 (58.3%)
Median Age (IQR)	43 (34–52)
Median Age at Onset (RIQ)	38.5 (29.3–46.5)
Median Disease Duration in Years (IQR)	5.0 (2.3–8.8)
Smoking (*n* = 82) (%)	6 (7.3%)
Comorbidities (*n* = 99) (%)	38 (38.38%)
Clinical Phenotype at Onset (%)	
Relapsing-Remitting MS (RRMS)	93 (90.29%)
Primary Progressive MS (PPMS)	8 (7.77%)
Clinically Isolated Syndrome (CIS)	1 (0.97%)
Secondary Progressive MS (SPMS)	1 (0.97%)
Current Clinical Phenotype (%)	
Relapsing-Remitting MS (RRMS)	82 (79.6%)
Primary Progressive MS (PPMS)	8 (7.8%)
Clinically Isolated Syndrome (CIS)	1 (1.0%)
Secondary Progressive MS (SPMS)	12 (11.7%)
Cerebrospinal Fluid (CSF) OCB positivity at Onset (%) (*n* = 49)	34 (63.4%)
Abnormal Evoked Potentials at Onset (%) (*n* = 46)	32 (69.6%)
Abnormal Evoked Potentials at Last Evaluation (%) (*n* = 43)	33 (76.7%)
Median Relapses Per Year (IQR)	0.6 (0.3–1.0)
Median Expanded Disability Status Scale (EDSS) Score at Onset (IQR)	
Relapsing-Remitting MS (RRMS)	3 (2–4)
Primary Progressive MS (PPMS)	-
Clinically Isolated Syndrome (CIS)	1 (1–1)
Secondary Progressive MS (SPMS)	-
Median EDSS Score at Last Visit (IQR)	
Less than 10 Years of Follow-up	
Relapsing-Remitting MS (RRMS)	3.5 (2.0–4.5)
Primary Progressive MS (PPMS)	4.0 (1.0–5.5)
Clinically Isolated Syndrome (CIS)	1.0 (1.0–1.0)
Secondary Progressive MS (SPMS)	5.5 (5.0–6.0)
Greater or equal than 10 Years of Follow-up	
Relapsing-Remitting MS (RRMS)	3 (2.5–4.8)
Primary Progressive MS (PPMS)	-
Clinically Isolated Syndrome (CIS)	-
Secondary Progressive MS (SPMS)	5 (4.0–6.0)

IQR: Interquartile Range.

**Table 2:** Clinical characteristics of the population at onset and at the last evaluation.

Characteristics	Onset	Last Evaluation
Motor Deficit	75/103 (72.8%)	81/103 (78.6%)
Spasticity	11/96 (11.5%)	61/102 (40.2%)
Optic Neuritis/Blurred Vision	42/100 (42.0%)	44/102 (43.1%)
Diplopia	27/98 (27.6%)	8/102 (7.8%)
Sensory Symptoms	75/103 (72.8%)	74/103 (71.8%)
Ataxia	33/101 (32.7%)	40/102 (39.2%)
Bladder Dysfunction	8/100 (8.0%)	21/102 (20.6%)
Constipation	7/101 (6.9%)	15/102 (14.7%)
Cognitive Dysfunction	8/90 (8.9%)	23/91 (25.3%)
Depression	19/94 (20.2%)	33/95 (34.7%)
Fatigue	28/94 (29.8%)	36/98 (36.7%)
Sexual Dysfunction	2/66 (3.0%)	11/60 (18.3%)
Facial Paresis	19/98 (19.4%)	13/102 (12.8%)
Abnormal Thermal Sensitivity	17/95 (17.9%)	13/101 (12.9%)
Lhermitte’s Sign	13/97 (13.4%)	7/100 (7.0%)
Paroxysmal Symptoms	19/102 (18.6%)	18/102 (17.7%)
Trigeminal Neuralgia	7/100 (7.0%)	5/103 (4.9%)
Facial Myokymia	5/100 (5.0%)	5/103 (4.9%)

**Table 3:** Treatments at disease onset and at last evaluation *.

Treatments	Onset (*n* = 103)	Last Evaluation (*n* = 103)
Moderately to highly effective therapies		
Ocrelizumab	2 (1.9%)	12 (11.7%)
Fingolimod	1 (0.9%)	6 (5.8%)
Low-efficacy therapies		
Teriflunomide	1 (0.9%)	1 (0.9%)
Glatiramer acetate	1 (0.9%)	0 (0.0%)
Interferon beta	46 (44.7%)	35 (34.0%)

* Only 51 (49.5%) participants reported some type of therapy at the onset of the disease and only 54 (52.4%) participants reported some type of therapy at the last evaluation.

## Data Availability

Data supporting the findings of this study are not publicly available due to ethical restrictions. The data were obtained from retrospective reviews of medical charts and include sensitive patient information; therefore, sharing is restricted to ensure confidentiality and compliance with institutional and ethical regulations.

## Abbreviations

The following abbreviations are used in this manuscript:

MS	Multiple sclerosis
RRMS	Relapsing-Remitting MS
PPMS	Primary Progressive MS
CIS	Clinically Isolated Syndrome
SPMS	Secondary Progressive MS
CRF	Case Record Form
IQR	Interquartile Range
